# Improving diagnostics using extended point-of-care testing during in-home assessments of older adults with signs of emerging acute disease: a prospective observational non-randomised pilot and feasibility study

**DOI:** 10.1186/s12877-024-04914-5

**Published:** 2024-04-25

**Authors:** Siri Aas Smedemark, Christian B. Laursen, Dorte Ejg Jarbøl, Flemming S. Rosenvinge, Karen Andersen-Ranberg

**Affiliations:** 1 Department of Geriatric Medicine, Odense University Hospital, Odense, Denmark; 2 Department of Clinical Research, University of Southern Denmark, Kløvervænget 2D, Indgang 112, 7. Sal, Odense, 5000 Denmark; 3 Department of Respiratory Medicine, Odense University Hospital, Odense, Denmark; 4 Research Unit of General Practice, Department of Public Health, University of Southern Denmark, Odense, Denmark; 5 Department of Clinical Microbiology, Odense University Hospital, Odense, Denmark; 6 Research Unit of Clinical Microbiology, University of Southern Denmark, Odense, Denmark

**Keywords:** Point-of-care testing, Focused lung ultrasound, Primary care, Geriatric, In-home assessments

## Abstract

**Background:**

Delayed recognition of acute disease among older adults hinders timely management and increases the risk of hospital admission. Point-of-Care testing, including Focused Lung Ultrasound (FLUS) and in-home analysis of biological material, may support clinical decision-making in suspected acute respiratory disease. The aim of this study was to pilot test the study design for a planned randomised trial, investigate whether in-home extended use of point-of-care testing is feasible, and explore its’ potential clinical impact.

**Methods:**

A non-randomised pilot and feasibility study was conducted during September–November 2021 in Kolding Municipality, Denmark. A FLUS-trained physician accompanied an acute community nurse on home-visits to citizens aged 65 + y with signs of acute respiratory disease. The acute community nurses did a clinical assessment (vital signs, capillary C-reactive protein and haemoglobin) and gave a presumptive diagnosis. Subsequently, the physician performed FLUS, venipuncture with bedside analysis (electrolytes, creatinine, white blood cell differential count), nasopharyngeal swab (PCR for upper respiratory pathogens), and urine samples (flow-cytometry). Primary outcomes were feasibility of study design and extended point-of-care testing; secondary outcome was the potential clinical impact of extended point-of-care testing.

**Results:**

One hundred consecutive individuals were included. Average age was 81.6 (SD ± 8.4). Feasibility of study design was acceptable, FLUS 100%, blood-analyses 81%, PCR for upper respiratory pathogens 79%, and urine flow-cytometry 4%. In addition to the acute community nurse’s presumptive diagnosis, extended point-of-care testing identified 34 individuals with a condition in need of further evaluation by a physician.

**Conclusion:**

Overall, in-home assessments with extended point-of-care testing are feasible and may aid to identify and handle acute diseases in older adults.

## Background

The population of older adults is increasing, and healthcare sectors worldwide face capacity challenges [1]. In Denmark, acute community healthcare services (ACHCS) were established in 2018 to carry out initial in-home clinical assessments of vulnerable citizens suspected of emerging acute diseases. The purpose was to support early decision-making and triage to reduce the number of avoidable admissions and the pressure on the secondary healthcare sector [2]. However, diagnosing older adults is challenging as they may present with vague symptoms, e.g., coughing is a less prominent symptom in pneumonia [3], or atypical symptoms e.g., functional decline, delirium, and falls [4, 5]. Delayed recognition of disease prevents timely management and increases the risk of hospital admission [6].

Point-of-care testing (POCT) is carried out bedside or near the patient, i.e., in-home [7], and increases timely diagnosis and decision-making in emergency departments and in primary care [8]. C-Reactive Protein (CRP), haemoglobin, international normalised ratio (INR), urine test strips, and blood glucose testing are widely implemented in primary care [9]. In recent years, new POCTs have been developed, such as white blood cell (WBC) differential count, hand-held point-of-care ultrasound, and urine flow-cytometry, but the tests are still not widely implemented in primary care nor validated among older adults [10–12]. The Danish ACHCSs use POCT for CRP and haemoglobin on capillary blood, but given the challenges of diagnosing older adults, a comprehensive approach is needed with additional clinical assessment, biochemical results, and imaging modalities [6]. By introducing extended POCT (ExtPOCT) during in-home assessment, we hypothesize that ExtPOCT improves diagnostic work-up and supports the primary care physicians’ clinical decision-making.

Prior to a planned randomised controlled trial (RCT), the primary objective was to investigate whether ExtPOCT during in-home assessments among older adults was feasible, and, secondly, to pilot-test the study design including the intervention consisting of ExtPOCT, defined by Focused Lung Ultrasound (FLUS) and in-home analysis of biological material (blood, nasopharyngeal swab, urine).

The secondary objective was to explore whether ExtPOCT had potential clinical impact by identifying conditions in need of clinical decision-making not identified by usual in-home assessments.

## Methods

### Trial design

This study was conducted as a prospective observational non-randomised pilot and feasibility study, adhering to the guidelines outlined by the CONSORT 2010 statement: extension to randomised pilot and feasibility studies [13, 14].

### Study setting

The study was conducted in 2021 from September 1^st^ to December 1^st^, in Kolding Municipality, Denmark, covering an area of 604.5 km^2^ with 93,161 inhabitants (65 + year olds: 18,453) [15]. The pilot study was conducted in collaboration with the ACHCS in Kolding Municipality.

The ACHCS is operated by acute community nurses (ACNs) trained in in-home assessment including vital signs and POCT for C-Reactive Protein (CRP) and haemoglobin on capillary blood (*usual* care) [2]. All clinical information is communicated to the primary care physician (PCP) to support clinical decision-making. In-home assessment is performed after referral from PCPs or home care service personnel when an acute condition in vulnerable citizens is suspected and can be carried out at the place of residence, i.e., in own home, care home, or skilled nursing facility. Approximately five patients are referred each day to the ACHCS for an in-home assessment. Hospital physicians refer patients for in-home treatment with intravenous antibiotics carried out by ACNs.

### Study participants

Participants eligible for this study were adults aged 65 years or older, referred to the ACHCS in Kolding Municipality for an acute in-home assessment, irrespective of their status as home care recipients or their place of residence, including own home, a care home, or a skilled nursing facility.

The participants had at least one of the following inclusion criteria: Cough, dyspnoea, fever (≥ 38 °C), chest pain, fall, or functional decline, defined as either subjective (not able to perform normal daily activities) or objective functional decline (increased need of home care service). Fall and functional decline are usually not perceived as symptoms of worsened or acute respiratory disease, but are known as atypical disease presentations [4, 5]. Participants with known moderate to severe cognitive impairment were excluded from the study, due to Danish legislation and recommendations from the Regional Committees on Health Research Ethics for Southern Denmark.

### Study size

A convenience sample of 100–150 participants was chosen to investigate feasibility and potential clinical impact. The pilot-study should not exceed 3 months, as inclusion rate was part of the feasibility assessment.

### Intervention

**Fig. 1:**
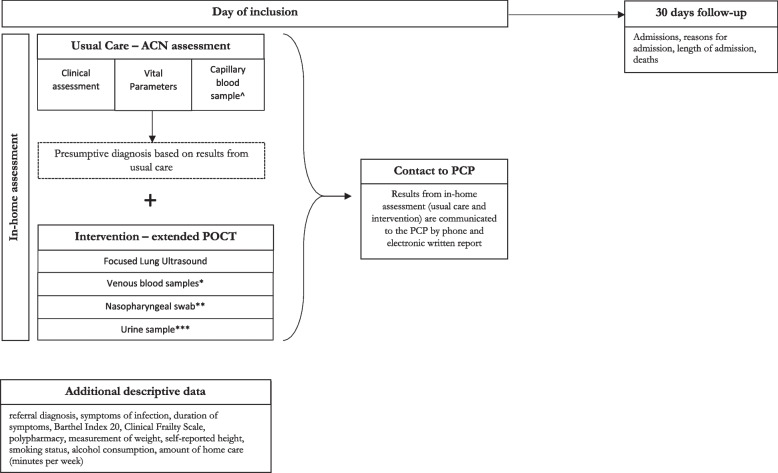
Overview of examination program and data collection. ^ POCT on capillary blood samples for C-reactive protein and Hemoglobin (using Quick-read PRO). *POCT on venous blood samples for creatinine and electrolytes (using i-STAT ®), and for Leucocytes with differential count (using Hemocue® WBF DIFF System). **POCT on nasopharyngeal swabs for 22 different viral and bacterial pathogens (using BioMérieux BioFire^®^FilmArray^®^ Respiratory Panel 2.1). ***POCT on urine samples for flow-cytometry (using Sysmex UF-5000 ®). Abbreviations: ACN: Acute Community Nurse, POCT: Point-of-care Testing, PCP: Primary Care Physician, PCR: Polymerase chain reaction

The intervention was an add-on to the ACNs’ usual in-home assessment and included hand-held FLUS, biochemical analysis on venous blood samples, and microbiological analysis of nasopharyngeal swabs and urine samples (Fig. 1).

FLUS examination was performed using a hand-held ultrasound scanner in the form of a Lumify® C5-2 Curved Array Transducer (Philips Medical Systems, Bothell, WA) (5–2 MHz, scan depth up to 30 cm) connected by USB-C to a FuturePAD® FPZ10-A tablet (CONCEPT International GmbH, Munich Germany). The standard Philips Lumify App version 4.0.1 software and its dedicated lung-preset was installed on the tablet and used for the examination. FLUS followed a standardised 14 scanning zone protocol using predefined questions regarding pneumothorax, pleural effusion, interstitial syndrome, and other obvious pathology [16, 17].

Blood-samples were collected by venipuncture and analysed immediately during in-home assessment. Blood samples for creatinine and electrolytes (collected in lithium/heparin vacutainers) were analysed using CHEM8+ cassettes on i-STAT® (Abbott, Inc., NJ, U.S.A.). WBC differential count (collected in EDTA-vacutainers) was analysed using HemoCue® WBF DIFF System (HemoCue AB, Ängelholm, Sweden) with dedicated micro-cuvettes.

Nasopharyngeal swabs and urine samples were collected during in-home assessment, and carried to Hospital Lillebaelt, Kolding, within 2 h. Nasopharyngeal swabs were analysed on the BioMérieux BioFire^®^FilmArray^®^ Respiratory Panel 2.1 (RP2.1) (BioFire Diagnostics, Salt Lake City, UT, USA). The BioFire^®^ FilmArray^®^ RP2.1 targets 22 respiratory pathogens [18]. Urine samples were analysed on urine flow-cytometer Sysmex UF-5000 ® (Sysmex Corporation, Kobe, Japan).

### Outcomes

Feasibility was evaluated based on several criteria, including the average inclusion rate, acceptability of the intervention, and the utilization of ExtPOCT. The average inclusion rate was considered feasible if at least two participants a day, 4 days a week, were enrolled in the study. The acceptability of the intervention was determined by assessing the number participants who declined participation and by assessing the acceptance of the results from ExtPOCT by PCPs. Additionally, the feasibility of ExtPOCT and collection of other biological material during in-home assessment was defined and calculated as the percentage of completed examinations. The intervention procedure (ExtPOCT) was considered feasible if used in > 80% of participants.

To assess the potential clinical impact of ExtPOCT, we predefined specific conditions in participants that necessitated clinical decision-making by the PCP based on usual care results, and subsequently compared these with the findings from ExtPOCT. Conditions requiring clinical decision-making were defined as instances necessitating treatment initiations or adjustments (e.g., antibiotics, diuretics, or inhalation therapy), hospital admission, or increased need for home care. The identification of participants with conditions requiring further clinical decision-making, which were not detected by usual in-home assessment conducted by ACNs, was considered indicative of potential clinical impact of ExtPOCT.

The registration of conditions requiring clinical decision-making was derived by using predefined cut-offs and international standardised agreements [16, 17, 19–22]. Conditions identified by ExtPOCT were: Pneumothorax, pleural effusion, interstitial syndrome, pneumonia, elevated leucocytes (> 11*10^9^/L), elevated creatinine (> 150 µmol/L), abnormal electrolytes (K^+^: < 3 mmol/L or > 5 mmol/L, Na^+^: < 125 mmol/L or > 145 mmol/L), positive PCR for upper respiratory tract pathogens, positive urine flow-cytometry (> 10^5^ BACT/ml).

### Data collection

An overview of the data collection and examination program is illustrated in Fig. 1.

The primary investigator, a physician certified and trained in thoracic ultrasound corresponding to European Federation of Societies for Ultrasound in Medicine and Biology (EFSUMB) thoracic ultrasound competency level 1, accompanied the ACNs during in-home assessment [23]. The *usual* in-home assessment was made by the ACNs, after which the ACNs noted a presumptive diagnosis and whether a clinical decision by a physician was required. Subsequently the primary investigator performed ExtPOCT.

All results from the usual care and ExtPOCT were communicated by the ACN to the participants PCP both by telephone and by written electronic communication.

The primary investigator collected descriptive data from all study participants during the in-home assessment (Fig. 1), including symptoms, symptom duration, height, smoking status, and alcohol consumption. Functional level was assessed using the Barthel Index 20, while frailty among participants was evaluated using the Clinical Frailty Scale, both of which were assessed using validated Danish-translated assessment scales [24, 25]. Additional data on the amount of home care received by participants was extracted from the municipal Electronic Social Care Record. Polypharmacy, defined as the use of 5 or more medications per day, was assessed using the Shared Medication Record (In Danish: FMK – Fælles Medicin Kort), a nationwide digital database at the Danish Health Data Authority, storing data on all Danish citizens’ current medication plans, electronic prescriptions, and medicine purchases [26].

The hospital Electronic Patient Journal (EPJ) was accessed after 30 days to register admissions, reasons for admission, length of admission, and deaths, as these variables are primary and secondary outcomes for the planned RCT. Adverse events and harms were also registered and served as safety assessment of the intervention.

### Statistical methods

We used descriptive statistics to present demographic and baseline characteristics. Categorical data was reported as number and percentage. Continuous data was reported as means (SD), medians [IQR], and range. The primary outcome was assessed by calculating inclusion-rate, number of declining participants, and number of contacts to the PCPs. The feasibility of ExtPOCT was calculated as the percentage of completed examinations. The secondary outcome was reported as the number of participants in need of clinical decision-making not identified by the ACNs usual in-home assessment. All statistical analyses were carried out using STATA version 16 software (StataCorp LLC, Texas, USA).

## Results

### Inclusion-flow

**Fig. 2:**
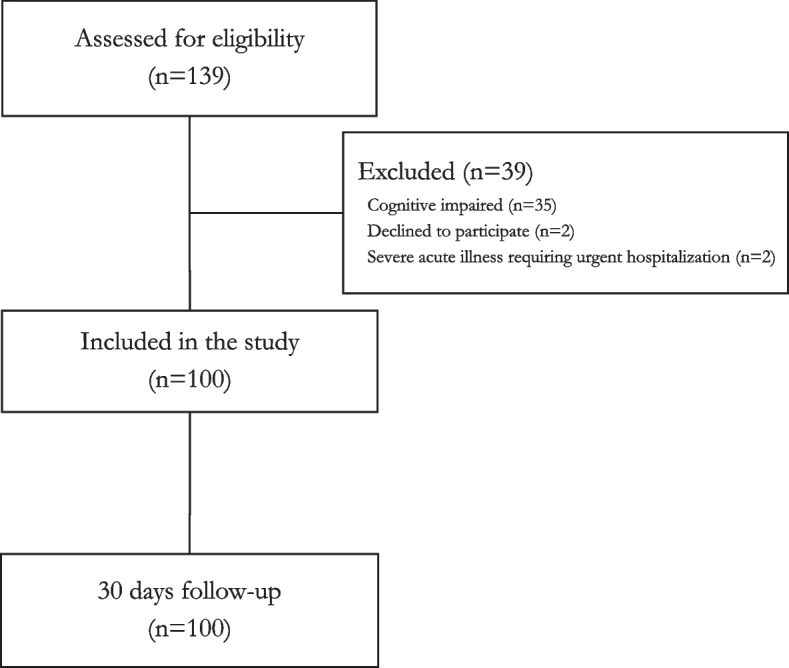
Flow-chart

Totally, 139 older adults were assessed for eligibility during the study period, of which 35 could not be included due to cognitive impairment, two declined, and two required urgent hospitalisation, thereby including 100 participants in the study. For details, see Fig. 2. Inclusion rate was 2.08 participants per day, 4 days a week, for 3 months. All participants were followed for 30 days.

### Study population

Mean age of included participants was 81.6 years (SD ± 8.4) and 54% were female. Most assessments were carried out in the participants own home (86%), of whom 71% received home care. The prevalence of polypharmacy (> 5 medications daily) was high (95%). Median Clinical Frailty Scale level was 5 (range 1–9). Less than four participants had chest pain. For details, see Table 1.

**Table 1:** Characteristics of study sample

**Characteristic**	**Total study population (*N* = 100)**
**Age, mean (SD)**	81.6 (8.4)
**Female, %**	54
**Place of in-home assessment, %**	
Own home	86
Care home	9
Skilled nursing facility	5
**Referred by, n**	
General practitioner	68
Home care	24
Other^a^	8
**Socioeconomic status, %**	
Living alone	60
Danish nationality	99
Daily alcohol intake	15
> 14 units/week	3
Smoking daily	23
Receiving home care^b^	71
**Polypharmacy, n**	
> 5 medications daily	95
**Functional level (Barthel 20)**	
Mean (SD)	14.9 (5.8)
**Clinical Frailty Scale (1–9)**	
Very fit, well, managing well (1–3)	26
Vulnerable (4)	19
Mildly Frail (5)	18
Moderately Frail (6)	15
Severely Frail (7)	12
Very Severely Frail (8)	3
Terminal Ill (9)	7
Median (IQR)	5 (3–6)
**Body Mass Index,**	
< 18	16
18–25	31
> 25–30	18
> 30	35
**Symptoms**	
Cough	52
Fever	23
Fatigue	61
Dyspnoea	68
Chest pain	3
Functional decline^c^	58
Other symptoms^d^	11
**Days ill**	
1 to 3 days	52
4 to 7 days	28
> 7 days	20

^a^Other: acute community nurses, nurses from care homes, palliative nurses

^b^Percentage of home care receivers among participants living in own home

^c^Functional decline defined as subjectively not able to conduct daily activities or objectively in need of increased home care

^d^Other symptoms: Abdominal pain, muscle pain, fall

### Usual in-home assessment

The ACNs carried out in-home assessment in all participants. Median values of vital signs are shown in Table 2. Median value of CRP and haemoglobin was 24 mg/L (IQR 5.3–57) and 7.4 mmol/L (IQR 6.6–8.1), respectively. ACNs presumptive diagnoses based on results from the usual in-home assessment suggested that 48 participants had conditions requiring clinical decision-making by a physician. The suggested diagnoses and conditions were pneumonia (*n* = 20), acute exacerbation of chronic obstructive pulmonary disease (*n* = 15), infection but unclear focus (*n* = 6), urinary tract infection (*n* = 5), and other (*n* < 3). For details, see Table 2.

**Table 2:** Findings from usual care and intervention

	**Total study sample (*N* = 100)**
**Usual care - ACNs in-home assessment**	
**Vital signs**	
Respiratory rate, breaths/min	
Median (IQR)	21(18–24)
Range	11–50
Saturation, %	
Median (IQR)	96(92–99)
Range	77–100
Systolic blood pressure, mmHg	
Median (IQR)	133 (117–143)
Range	81–208
Diastolic blood pressure, mmHg	
Median (IQR)	74 (67–80)
Range	49–113
Heart rate, beats/min	
Median (IQR)	81 (71–92)
Range	44–116
Body temperature	
Median (IQR)	36.9 (36.6–37.4)
Range	35.7–40
GCS	
Median (IQR)	15 (15–15)
**Simple POCT**	
C-Reactive protein, mg/L	
Median (IQR)	24 (5.3–57)
Range	0.6–171
Haemoglobin, mmol/L	
Median (IQR)	7.4 (6.6–8.1)
Range	0.1–10.2
**Presumptive diagnoses, made by the acute nurse, n**	
Pneumonia	20
Viral infection	18
Urine infection	5
Infection, unclear focus	6
AE-COPD^b^	15
Other^c^	36
**Intervention – Results from Extended POCT**	
**Feasibility of extended POCT, n**	
FLUS	100
Creatinine, electrolytes, leucocytes with differential count	89
PCR for upper respiratory pathogens	79
Urine flow cytometry	4
**FLUS, n**	100
**Patient positioning**	
8 scanning zones	28
14 scanning zones	72
**Normal**	36
**Pneumothorax**	≤ 3^a^
**Interstitial syndrome**	8
**Pleural Effusion**	32
Unilateral	25
Bilateral	≤ 3^a^
Simple	30
Complex	≤ 3^a^
Small	26
Moderate	3
Large	3
**Pneumonia, n**	25
Consolidated lung tissue	≤ 3^a^
Dynamic bronchoaerograms	≤ 3^a^
Focal B-lines	7
Focal B-lines, fragmented and thickened visceral pleura, and pleural effusion	15
**Other pathologies, n**	16
Thickened parietal pleura	7
Uncharacteristic lung consolidations	6
Tumour/suspected malignancy	3
**Venous blood samples, n**	89
**Creatinine, µmol/L**	
Median (IQR)	80 (66–118)
Range	24–281
**Na**^**+**^**, mmol/L**	
Median (IQR)	138 (136–140)
Range	126–144
**K**^**+**^**, mmol/L**	
Median (IQR)	4 (3.7–4.2)
Range	3.2–5.3
**Blood Urea Nitrogen (BUN), mmol/L**	
Median (IQR)	7.7 (6.2–10.5)
Range	1.8–26.5
**Total leucocytes, 10**^**9**^**/L**	
Median (IQR)	9.4 (7.3–12,1)
Range	1.6–25
**Neutrophils, 10**^**9**^**/L**	
Median (IQR)	5.1 (3.9–7.7)
Range	0.3–19
**Lymphocytes, 10**^**9**^**/L**	
Median (IQR)	2.3 (1.8–2.8)
Range	0.8–5.2
**Monocytes**, **10**^**9**^**/L**	
Median (IQR)	0.6 (0.4–0.7)
Range	0–1.6
**Eosinophils, 10**^**9**^**/L**	
Median (IQR)	0.3 (0.2–0.6)
Range	0–4
**Basophils, 10**^**9**^**/L**	
Median (IQR)	0 (0–0.1)
Range	0–0.9
**Biofire filmarray, n**	79
Respiratory pathogens detected	≤ 3^a^
**Urine samples, n**	4
> 10^5^ BACT/ml	≤ 3^a^
**Primary care physician contact**	
**Primary care Physician, n**	
Doctor reached by phone,	61
Secretary reached by phone	17
No phone contact	22
**Initiated treatment during in-home-assessments, n**	48
Antibiotic, oral, no (%)	18 (37)
Diuretics, oral, no (%)	3 (6)
Acute hospital admission following in-home assessments, no (%)	21 (43)
Other treatments^d^, no (%)	12 (25)
**Planned follow-up in-home assessment by ACN**	
Planned new in-home assessment in 3 days	15

*Abbreviations*: *ACN* Acute Community Nurse, *FLUS* Focused Lung Ultrasound, *POCT* Point-of-care Testing

^a^Due to Danish Legislation and EU General Data Protection Agency reporting of observations below 3 is not permitted

^b^*AE-COPD* Acute exacerbation of chronic obstructive pulmonary disease

^c^Other: suspected malignancy (6), functional decline (4), chronic condition (10), orthostatic hypotension (4), dehydration (≤ 3), heart problem (≤ 3), skin infection (≤ 3), deep venous thrombosis (≤ 3), not ill (≤ 3), anaemia (≤ 3), unclear but ill (≤ 3)

^d^Other treatments: Salbutamol inhalation (3), prednisolone (3), increased home care (3), subcutaneous furosemide injection (≤ 3), cutaneous antibiotic (≤ 3), change in antibiotics (≤ 3)

### Intervention – ExtPOCT

FLUS was performed in all participants (100%), venous blood-samples in 81%, nasopharyngeal swabs in 79%, and urine samples in 4%. Reasons for missing venous blood samples were practical difficulties in obtaining a blood sample. Reasons for missing nasopharyngeal swabs were acute admissions, and urine samples were missing due to permanent catheters (27%) or participants unable to provide a urine sample (73%) during the in-home assessment.

**Fig. 3:**
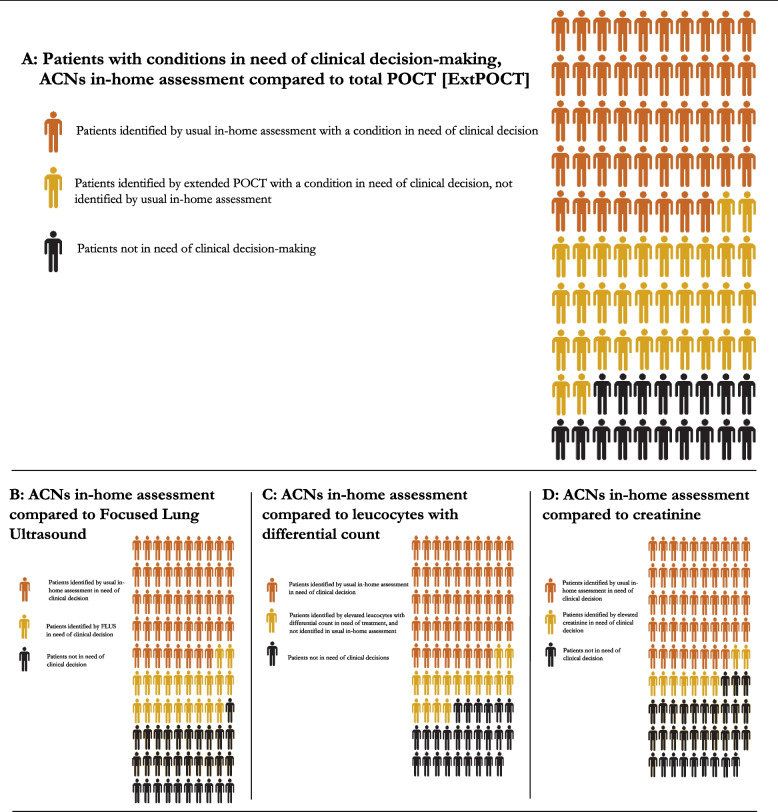
Potential clinical impact of extended point-of-care testing. Extended point-of-care testings’ potential for clinical impact by identifying additional participants with conditions in need of clinical decision-making compared to usual in-home assessments. **A** ACNs identified 48 out of 100 participants in need of clinical decision-making by a primary care physician. Extended POCT identified additional 34 participants with a condition in need of clinical decision-making, not identified by the usual in-home assessment. Each specific POCT is illustrated in **B**, **C**, **D**, except for PCR for upper respiratory tract infection and urine flow-cytometry, as results did not change clinical decision-making: **B** Focused Lung ultrasound identified 21 participants with a condition in need of clinical decision-making not identified by the ACNs in-home assessment. (Feasibility 100%). **C** POCT for leucocytes with differential count identified 16 participants with a condition in need of clinical decision-making not identified by the ACNs in-home assessment. (Feasibility 89%). **D** POCT for creatinine identified 9 participants with a condition in need of clinical decision-making not identified by the ACNs in-home assessment. (Feasibility 89%). Abbreviations: ACN: Acute Community Nurse, ExtPOCT: Extended point-of-care testing, FLUS: Focused Lung Ultrasound, POCT: Point-of-care Testing, PCR: Polymerase chain reaction

ExtPOCT identified additional 34 participants with acute conditions requiring clinical decision-making by a physician. This is illustrated in Fig. 3. FLUS alone identified additional conditions in 21 participants needing further clinical decision-making by a physician: Pneumothorax, interstitial syndrome, moderate to large pleural effusions, and pneumonia. A significantly elevated creatinine (> 150 µmol/L) supporting a diagnosis of dehydration was identified in nine participants, which was not identified in the usual in-home assessment. Elevated WBC differential counts were identified in 16 participants, not suspected to have a bacterial infection in the usual in-home assessment. PCR for respiratory pathogens identified less than three viral infections. Only few urine samples were collected, and the potential clinical impact is therefore uncertain.

### Healthcare contacts

In 62 of the in-home assessments, the PCP was contacted on the day of the visit directly by phone, and in 77% of the cases, the contact led to initiation of treatment, mainly oral antibiotics (37%), diuretics (6%), or hospital admission (21%). In total 42 participants (55 admissions) were admitted to hospital from the day of inclusion to 30 days follow-up. Median duration of hospital admission was 5 days (IQR 2–8).

### Adverse events

No adverse events were reported to or observed by the research team. In total, 11 deaths occurred, and most deaths took place in hospital (*n* = 7).

## Discussion

### Key results and interpretation

The overall study design and use of ExtPOCT during in-home assessment of older adults were feasible. Our results suggest that FLUS and POCT on venous blood might supplement the usual in-home assessment of older adults suspected of acute respiratory disease by identifying additional conditions - potentially facilitating diagnostic work-up and early treatment.

The study design of the planned RCT has been modified based on the insights gained from the pilot study. As a result, the inclusion criteria were revised, which involved omitting chest as an eligibility symptom. This adjustment was informed by the observation that very few participants presented with chest pain. It is also important to note that in Denmark, chest pain as a standalone symptom typically warrants acute admission regardless of other factors. Therefore, including chest pain as a criterion in our study was deemed potentially confounding, and it was decided to exclude chest pain as a specific inclusion criterion for our study. We also omitted nasopharyngeal swab for PCR for upper respiratory pathogens and urine flow-cytometry from the intervention as explained in the following sections.

FLUS had the highest feasibility and did provide additional information to the clinical decision making. These findings are in line with other studies, showing that FLUS identifies missed conditions in need of treatment [16, 27, 28]. In general, only few studies on the use of FLUS have been conducted in primary care [29]. A recent study conducted in primary care investigated lung ultrasound performed by PCPs in patients suspected of community-acquired pneumonia found a higher prevalence of pneumonia compared to our findings (53% vs 25%) [30]. However, they used more specific inclusion criteria for community-acquired pneumonia, and patients were younger (median age of 47 vs 81.6 years). Only one participant in our study had classic dynamic bronchoaerograms, whereas most participants with pneumonia had unspecific findings with a thickened, fragmented visceral pleura, smaller sub-pleural consolidations, and focal B-lines [19]. Another notable finding from FLUS was pleural effusions: The identified moderate to large pleural effusions were among individuals without classic respiratory symptoms, but functional decline. In conclusion, FLUS is highly feasible, could have clinical impact, and will remain part of the intervention in the planned RCT.

Elevated creatinine level led to initiation of treatment or hospital admission in cases not identified by usual in-home assessment. Elevated leucocytes > 11*10^9^/L were interpreted as sign of bacterial infection, although cut-off points on different biomarkers of inflammation in older multimorbid adults have not reached international consensus and/or are still not validated [10, 11, 31]. In addition, systematic reviews and prospective studies have shown that older adults differ in biochemical presentation compared to younger adults [32–34]. This highlights the need for further research in diagnosing infections in older adults. Due to high feasibility and potential clinical impact, we chose to keep both creatinine, electrolytes, and WBC differential count in the planned RCT.

Nasopharyngeal swabs were feasible to collect, but PCR for upper respiratory pathogens did not add to the clinical decision-making. During the study period, rates of viral respiratory tract infections were very low probably as a consequence of general restrictions and recommendations to reduce and contain the COVID-19 pandemic in Denmark [35]. Because of the possible low number of positive samples, the uncertain clinical impact, and the high cost, POCT PCR was omitted from the RCT.

Urine samples were difficult to collect as many participants had catheters or were unable to provide a urine sample during the in-home assessment. Hence, the added clinical value of urine analysis is unknown and urine flow-cytometry is therefore excluded from the RCT.

### Healthcare contacts

The high rate of hospital admissions during the 30 days follow-up highlights the need for early detection of suspected disease in older adults. ExtPOCT might have added to the high rate by detecting pathologies in need of hospital admissions e.g., large pleural effusions. Additionally, ExtPOCT may yield false positive findings, or unclear results leading to unnecessary contacts to the secondary healthcare sector. However, it also has potential for early, relevant treatment decisions to prevent clinical deterioration and subsequent functional decline. Our planned RCT aims to investigate the effect of ExtPOCT on specific healthcare outcomes such as hospital admissions, in-hospital length of stay, and mortality, thereby addressing the effect of ExtPOCT on hospital admissions.

POCT for CRP, haemoglobin, blood glucose, and urine test strips, are widely implemented in primary care, but there is limited evidence of using other POCT in primary care especially among older adults and during in-home assessments [10, 11, 36]. A recent study from Germany highlighted that many PCPs rated only a limited number of POCT as useful [37], but without explaining why. Barriers towards POCT among PCPs, e.g., low economic benefit, over-reliance, increased risk of over-treatment, over-diagnostics, and unnecessary hospital admissions, can hinder implementations. We therefore plan to explore how citizens, ACNs, and PCPs experience ExtPOCT during in-home assessment, in a user-perspective evaluation after completion of the planned RCT.

### Generalisability

The study sample was older adults with high rates of polypharmacy, frailty, and home-care dependency – a group of older adults who are at increased risk of hospital admission, and similar to the study population we aim to include for our planned RCT [1, 38].

The ACHCS setup is common in Scandinavia, though organised differently between countries and municipalities [39]. Results from the present pilot-study and the planned RCT might not be applicable in other countries than Denmark, but the concept is applicable: By increasing competencies, e.g., introducing in-home POCT, to healthcare professionals caring for frail older adults, timely clinical decisions may be facilitated.

### Limitations

Our study is classified as a pilot and feasibility study, as it aims to assess both the feasibility of the intervention and its potential clinical impact. Although our study lacked randomisation, we aimed to pilot-test the intervention and explore our hypothesis that ExtPOCT had potential to enhance the diagnostic work-up. Acceptability of the intervention was utilized as an outcome measure to assess feasibility. While acceptability traditionally focuses on participants’ and stakeholders’ attitudes towards an intervention, data collection process, or randomization, we followed the approach advocated by Eldridge et al. to explore acceptability as a means of informing the feasibility of a larger RCT [14]. Therefore, we utilized acceptability as a measure of feasibility to evaluate whether the intervention could be effectively implemented in a future RCT.

We had to exclude participants with cognitive impairment, and therefore we do not know whether ExtPOCT is feasible or have potential clinical impact in the diagnostic work-up among older adults with cognitive impairment.

We are well aware that it is not possible for us to determine the causal effect of ExtPOCT on clinical impact, and we do not have a final diagnosis on all participants. However, it was important to explore the potential clinical impact prior to the RCT: If the usual in-home assessments carried out by ACNs can identify most conditions in need of clinical decision-making, ExtPOCT is redundant.

The primary investigator performed ExtPOCT. Prior to the planned RCT, ACNs will complete an extensive training programme for collecting venous blood samples, handling POCT, and performing FLUS, and subsequently carry out ExtPOCT during the RCT.

## Conclusions

The overall study design for the planned RCT is feasible, and in-home blood analyses and FLUS have a potential clinical impact by identifying acute conditions earlier in the diagnostic process, which suggests a potential for improving clinical decision-making during in-home assessment among older adults.

## Data Availability

The dataset generated and analysed during the current study is available from the corresponding author on reasonable request.

## Abbreviations

ACHCS: Acute community healthcare service
CAN: Acute community nurse
CFS: Clinical Frailty Scale
CRP: C-reactive protein
ExtPOCT: Extended Point-of-care testing, including a focused lung ultrasound scan (FLUS), biochemical analysis on venous blood samples, and microbiological analysis of nasopharyngeal swabs and urine samples
FLUS: Focused lung ultrasound scan
GDPR: General Data Protection Regulation
INR: International Normalised Ratio
OPEN: Open Patient data Explorative Network
PCR: Polymerase chain reaction
POC: Point-of-care
POCT: Point-of-care testing
RCT: Randomised controlled trial
WBC: White blood cells
